# Supplementary material for: Actinotrichia-independent developmental mechanisms of spiny rays facilitate the morphological diversification of Acanthomorpha fish fins
Source: Nat Commun. 2026 Feb 14;17:2775. doi: 10.1038/s41467-026-69180-y (PMC13018208; doi:10.1038/s41467-026-69180-y)
Supplement: Supplementary file 1 — Supplementary Information [file 41467_2026_69180_MOESM1_ESM.pdf]

Supplementary information for

Actinotrichia-independent developmental mechanisms of spiny rays facilitate the morphological diversification of Acanthomorpha fish fins

Kazuhide Miyamoto, Junpei Kuroda, Satomi Kamimura, Yasuyuki Sasano, Gembu Abe, Satoshi Ansai, Noriko Funayama, Masahiro Uesaka and Koji Tamura

Corresponding author: Kazuhide Miyamoto

Email: kazuhide.miyamoto.t5@dc.tohoku.ac.jp

Supplementary Table 1. Micro-CT scanning conditions used in this study.

Specimen	Tube voltage peak	Tube current	Isotropic resolution	CT scanner
*Melanotaenia praecox*_1	100 kV	100 µA	19.317 µm	ScanXmate-D180RSS270
*Melanotaenia praecox*_2	100 kV	200 µA	18.81 µm	VR150V-HH-TAVE
*Melanotaenia praecox*_3	100 kV	200 µA	18.81 µm	VR150V-HH-TAVE
*Stephanolepis cirrhifer*_1 (whole body)	100 kV	70 µA	7.300 µm	ScanXmate-D180RSS270
*Stephanolepis cirrhifer*_1 (close-up view)	100 kV	50 µA	4.840 µm	ScanXmate-D180RSS270
*Stephanolepis cirrhifer*_2 (whole body)	50 kV	100 µA	4.55 µm	VR150V-HH-TAVE
*Stephanolepis cirrhifer*_2 (close-up view)	50 kV	100 µA	2.12 µm	VR150V-HH-TAVE

Supplementary Table 2. Primer sequences used in this study.

Name	Sequence (5′–3′)	Usage
Mpraecox_and1_fw1	TTTGCTTCTCCTCCAGAAGC	Cloning
Mpraecox_and1_fw2	GCCTGGGTTAGTATGTATCTGC	Cloning & genotyping
Mpraecox_and1_rv1	AGGACCGTGTATTCATGTCC	Cloning
Mpraecox_and1_fw3	GGGTGAAAGTATCCAGGAGAAGTC	Cloning & making probes
Mpraecox_and1_rv2	GAAGGGATCATTAGGGTTAGGCTC	Cloning & making probes
Mpraecox_and2_fw1	ATTACTATGCTCCAGTCCTGGAGC	Cloning & genotyping
Mpraecox_and2_rv1	GAGTGAGCACCATAACTACCAG	Cloning
Mpraecox_and2_fw2	GATCGTCTGTACATGGCCTACTTG	Cloning, genotyping & making probes
Mpraecox_and2_rv2	GCAGTTGTAGTCATACTCAGGGTC	Cloning & making probes
Mpraecox_and1_gen_fw1	GGGTGAAAGTATCCAGGAGAAGTC	Genotyping
Mpraecox_and1_gen_rv1	ATAATATCCTGACTGTGGGTCCTTGGCAGGAACTGC	Genotyping
Mpraecox_and1_gen_rv2	TCAGTGAAGTACTTGTACCAGGCC	Genotyping
Mpraecox_and2_gen_rv	ATCATATGGATGGCAGAAGGGTGC	Genotyping
Mpraecox_and2_gen_rv2	TTGCAGTGCTGCTCTTTAGG	Genotyping

Supplementary Table 3. CRISPR target sequences in this study.

sgRNA_Name	Direction	Sequence (5′–3′)
sgRNA-and1#1	Sense	CCGCTTTGCACAGGAGGCTGATC
	Anti-sense	GATCAGCCTCCTGTGCAAAGCGG
sgRNA-and1#2	Sense	CCCAGCTCCACCACCAGCAACCC
	Anti-sense	GGGTTACTGGTGGTGGAGCTGGG
sgRNA-and2#1	Sense	CCCCCCCTGGTCAAAAGGGCCCC
	Anti-sense	GGGGCCCTTTTGACCAGGGGGGG
sgRNA-and2#2	Sense	CGGAGTCCGCGGCAAGACCAAGG
	Anti-sense	CCTTGGTCTTGCCGCGGACTCCG

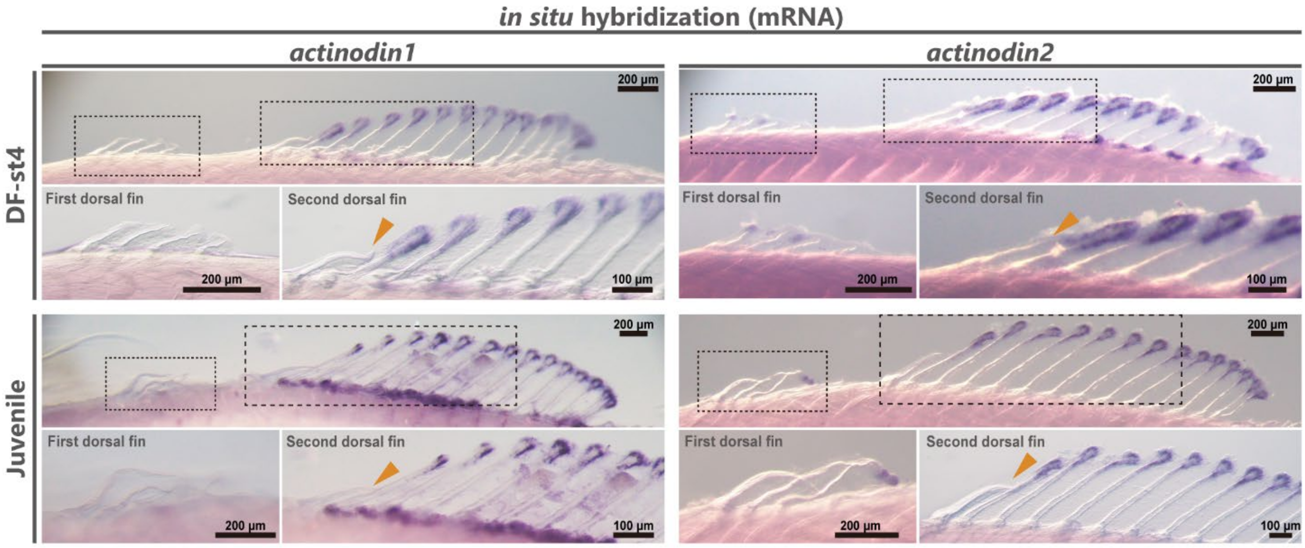

Supplementary Figure 1. *actinodin1* and *actinodin2* gene expression in the dorsal fin of *Melanotaenia praecox.*

In situ hybridization at the DF-st4 and juvenile stages, showing *actinodin1* and *actinodin2* expression in the first and second dorsal fins (*and1*, DF-st4 larvae, $n = 6$; *and2*, DF-st4 larvae, $n = 4$; *and1*, juvenile, $n = 4$; *and2*, juvenile, $n = 4$). Orange arrows indicate the spiny-ray in the second dorsal fin. Magnified image of the first- and second dorsal fin area marked by the black dashed box.

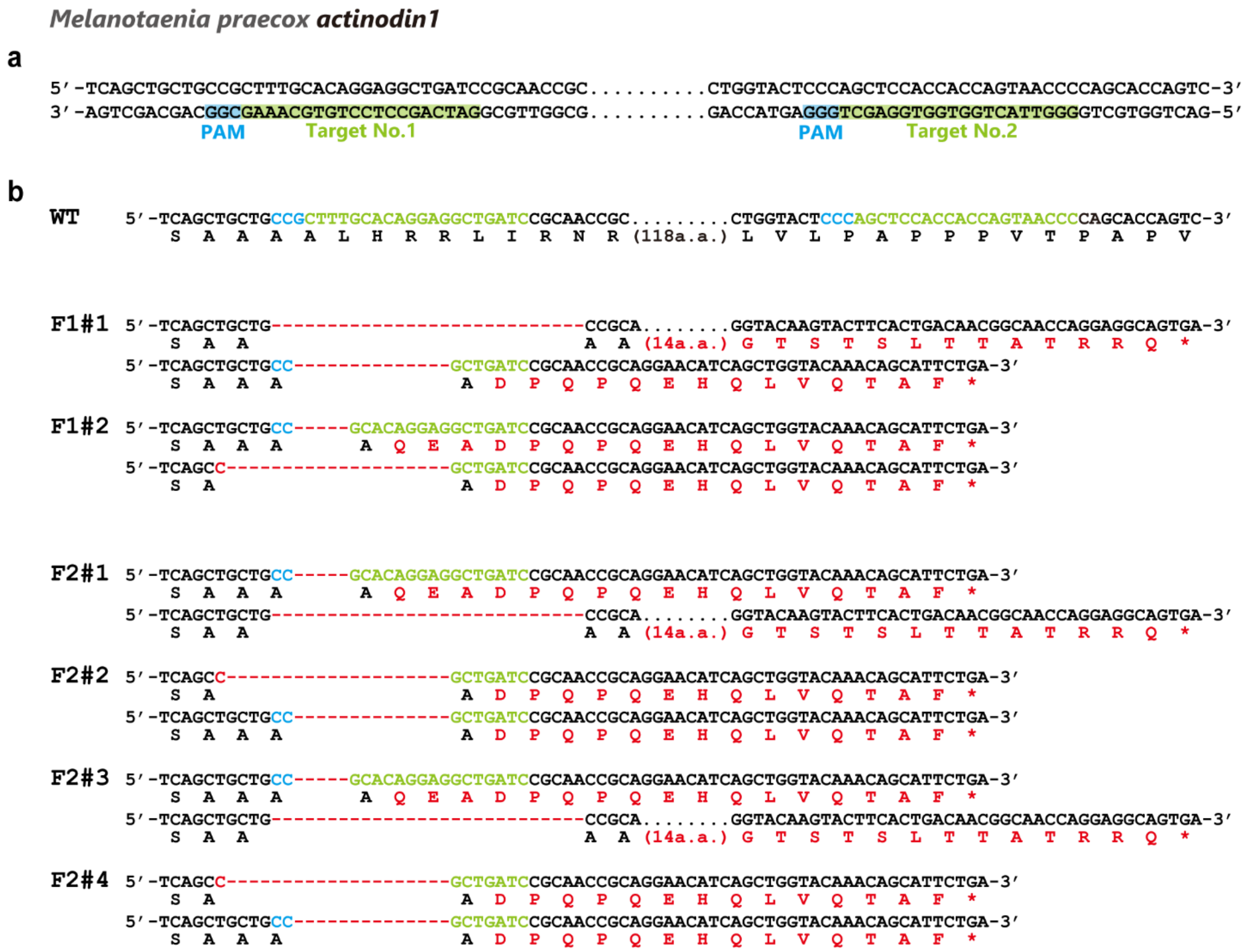

Supplementary Figure 2. Targeted knockout of the *actinodin1* gene in *Melanotaenia praecox* using the CRISPR/Cas system.

a Schematic illustration of the design of sgRNAs (see Supplementary Table 3) targeting the *actinodin1* (*and1*) gene. The nucleotide sequences marked by blue and green boxes are the protospacer adjacent motif (PAM) and the recognition sequence for the sgRNA target, respectively. **b** Sequences of wild-type (WT), F1, and F2 embryos, showing a mutation spectrum revealed by Sanger sequencing. Red dashes indicate identified deletions; sequences in blue and green indicate the PAM and the recognition sequence for the sgRNA target, respectively. The sample numbers are shown to the left of each sequence. a.a., amino acids.

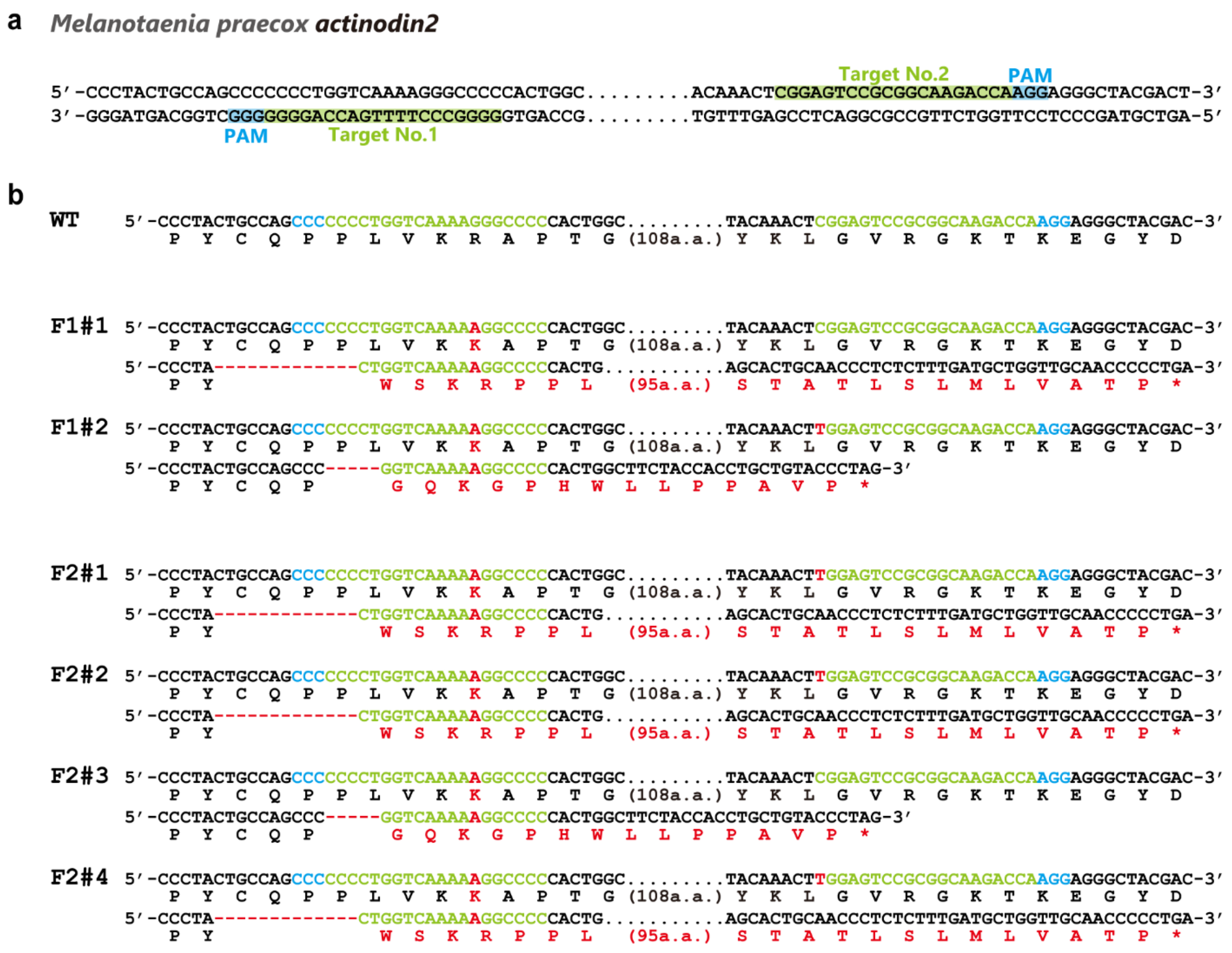

Supplementary Figure 3. Targeted knockout of the *actinodin2* gene in *Melanotaenia praecox* using the CRISPR/Cas system.

a Schematic illustration of the design of sgRNAs (see Supplementary Table 3) targeting the *actinodin2* (*and2*) gene. The nucleotide sequences marked by blue and green boxes show the protospacer adjacent motif (PAM) and the recognition sequence for the sgRNA target, respectively. **b** Sequences of wild-type (WT), F1, and F2 embryos, showing a mutation spectrum revealed by Sanger sequencing. Red dashes indicate identified deletions; sequences in blue and green indicate the PAM and the recognition sequence for the sgRNA target, respectively. The sample numbers are shown to the left of each sequence. a.a., amino acids.

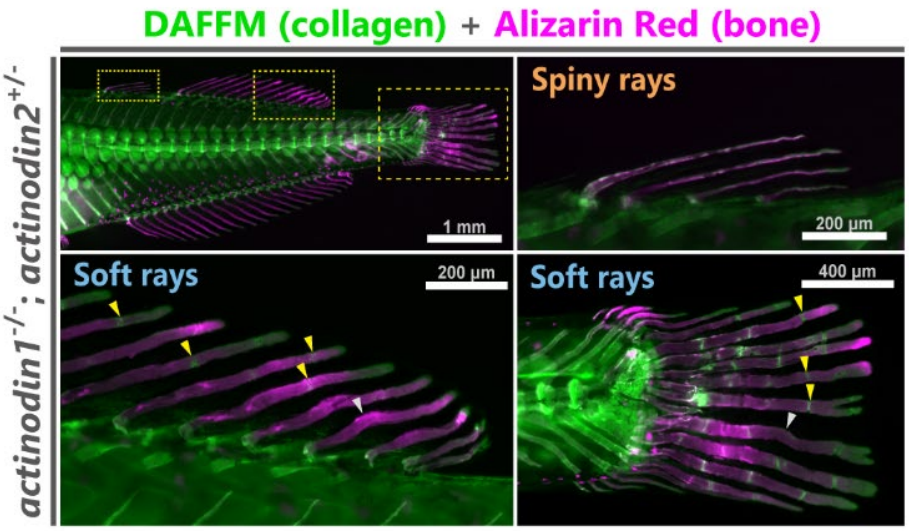

Supplementary Figure 4 Actinotrichia distribution and spiny- and soft-ray bone morphology in the *actinodin1*$^{-/-}$/*actinodin2*$^{+/-}$ knockout *Melanotaenia praecox*.

White arrowheads indicate abnormal bending of the soft rays in the knockout fish. Yellow arrow heads show aberrantly positioned segmental structures in the soft rays of the knockout fish. Actinotrichia were labeled with DAFFM DA (green), and the spiny- and soft-ray bones were labeled with alizarin red (magenta). All panels except the top-left panel represent magnified views of the area enclosed by the yellow dashed box in the top-left panel.

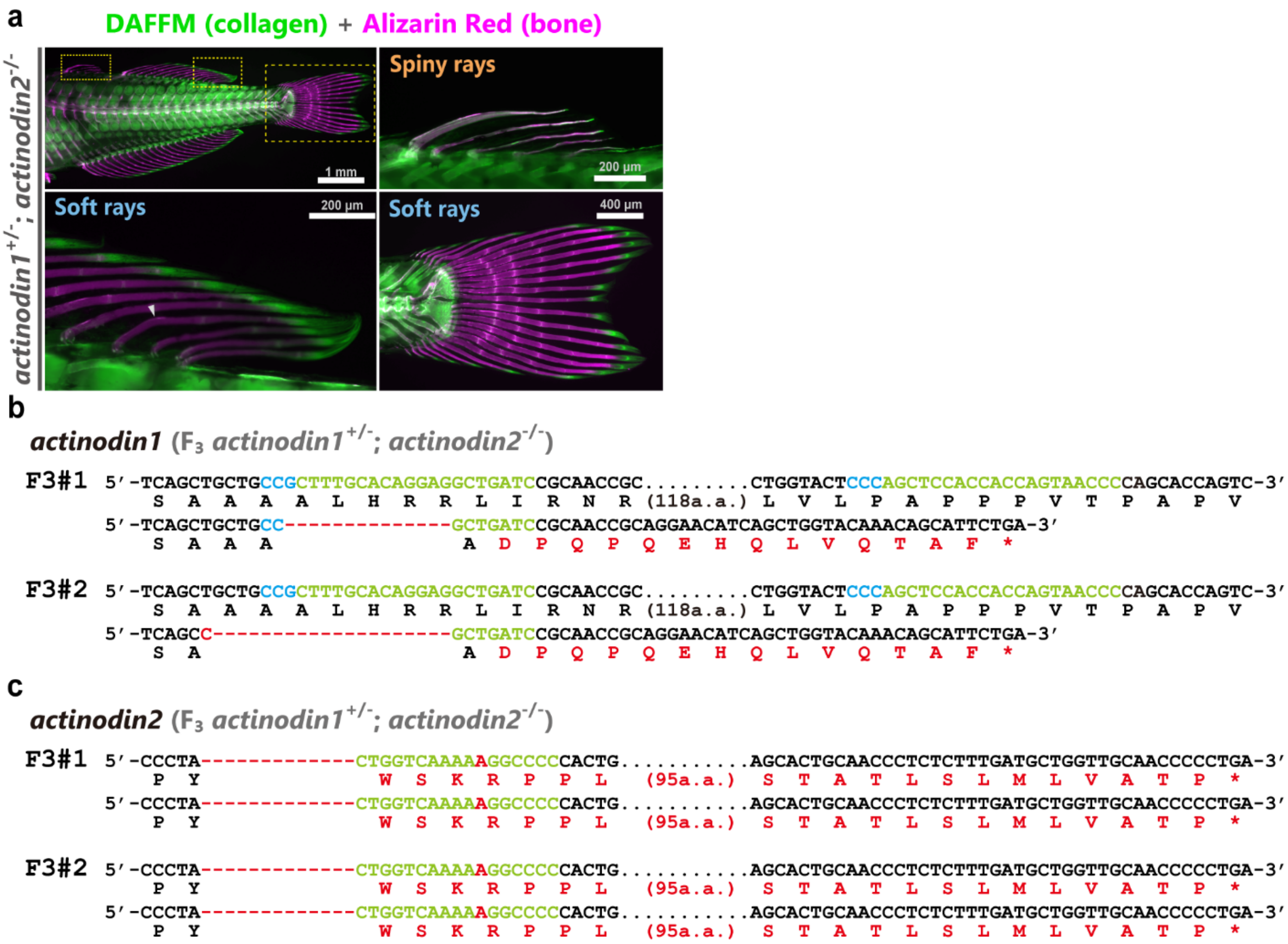

Supplementary Figure 5 Actinotrichia distribution and spiny- and soft-ray bone morphology in the *actinodin1*$^{+/-}$/*actinodin2*$^{-/-}$ knockout *Melanotaenia praecox*.

a White arrowheads indicate abnormal bending of the soft rays in the knockout fish (n = 2). Actinotrichia were labeled with DAFFM DA (green), and the spiny- and soft-ray bones were labeled with alizarin red (magenta). All panels except the top-left panel represent magnified views of the area enclosed by the yellow dashed box in the top-left panel. **b-c** Sequences of F3 embryos, showing a mutation spectrum revealed by Sanger sequencing. Red dashes indicate identified deletions; sequences in blue and green indicate the PAM and the recognition sequence for the sgRNA target, respectively. The sample numbers are shown to the left of each sequence. a.a., amino acids.

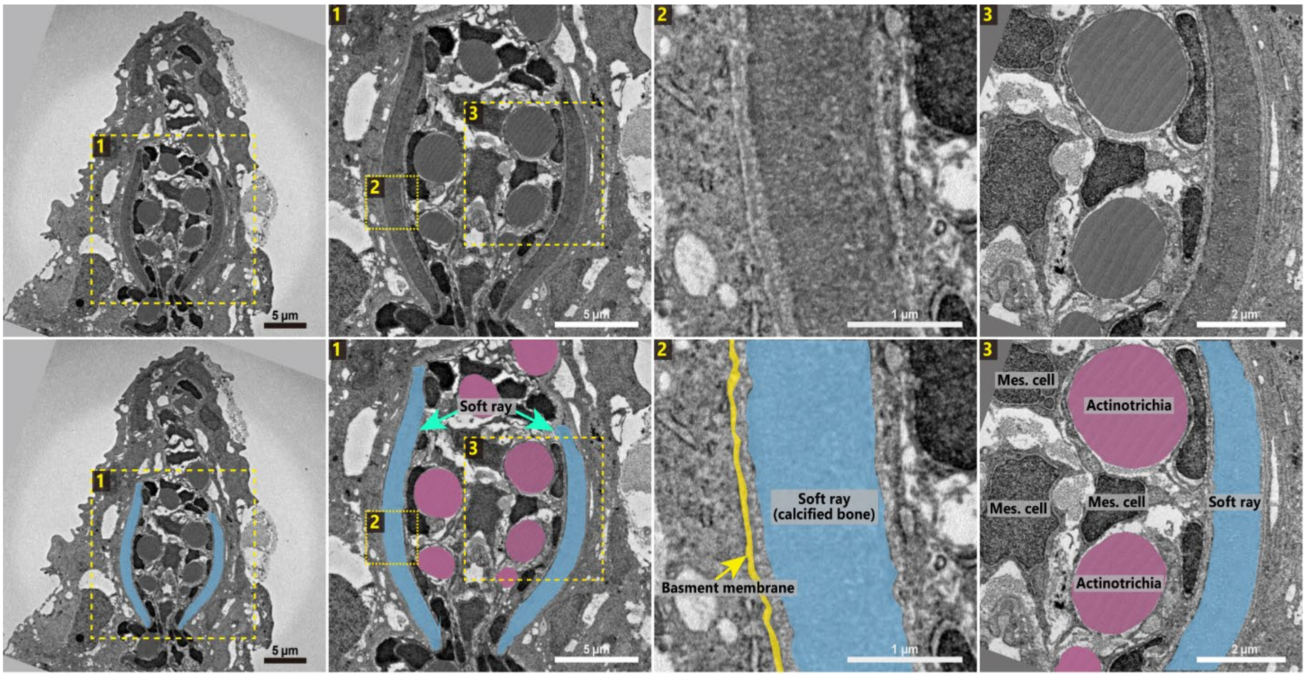

Supplementary Figure 6. Ultrastructural analysis of soft rays in *Melanotaenia praecox* by TEM. Sections of the second dorsal fin showing the structural organization of the soft-ray tip in larvae at DF-st4 (*n* = 3). The same TEM images are presented corresponding to the lower panels, with the soft ray (light blue), actinotrichia (magenta), and basement membrane (yellow) highlighted in different colors. The numbers of each panel (#1, #2, #3) correspond to the numbers in yellow dashed boxes. Mes, mesenchymal.

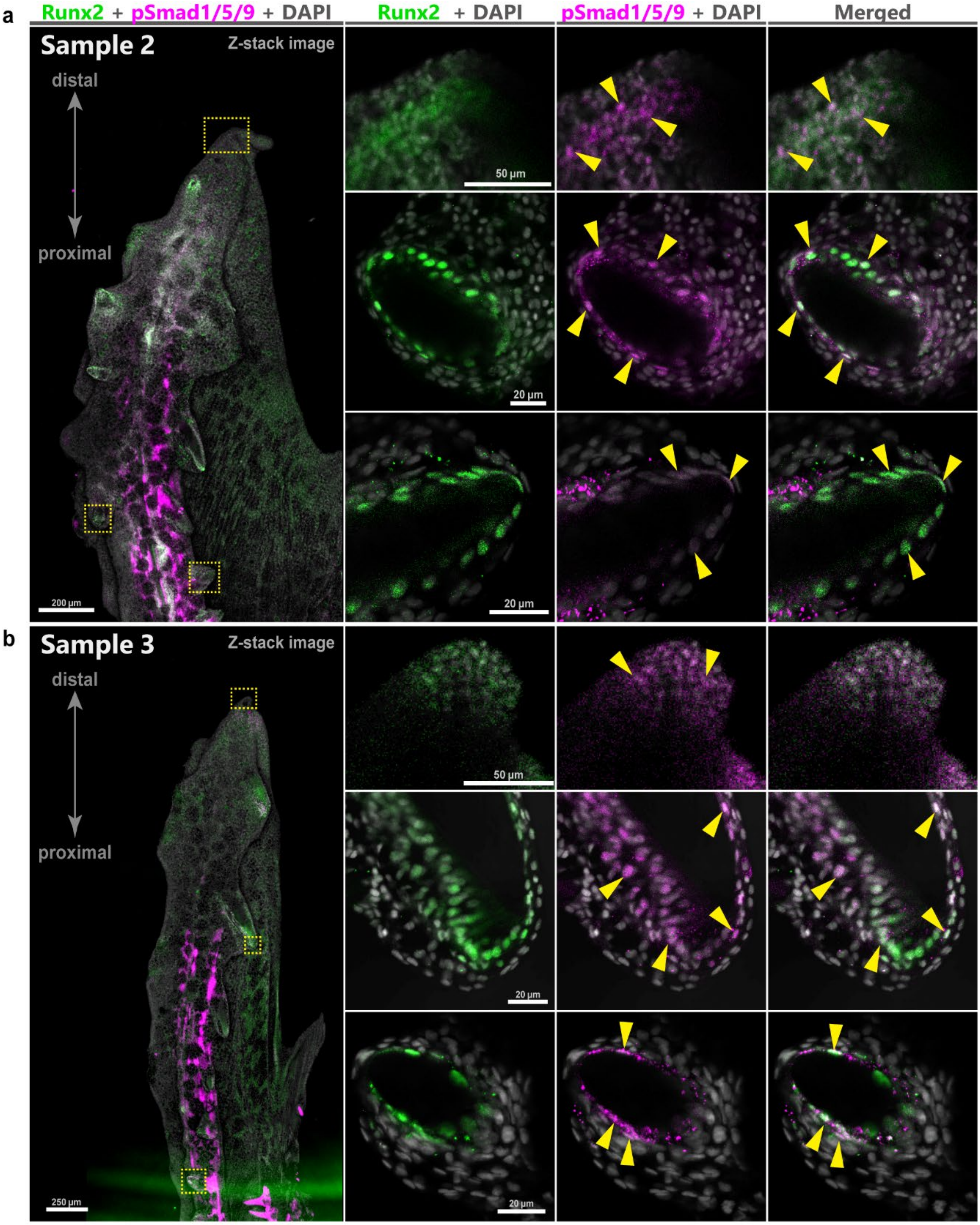
a
Runx2 + pSmad1/5/9 + DAPI
Runx2 + DAPI
pSmad1/5/9 + DAPI
Merged
Sample 2
Z-stack image
distal
proximal
200 µm
50 µm
20 µm
20 µm
b
Sample 3
Z-stack image
distal
proximal
250 µm
50 µm
20 µm
20 µm

Supplementary figure 7. Premature osteoblasts with BMP signaling are spatially modified from the tip to the lateral protrusions of the first dorsal-fin spiny ray in *Stephanolepis cirrhifer*.

a, b Distributions of Runx2-positive cells (green) and pSmad1/5/9-positive cells (magenta) in the dorsal spine of two *S. cirrhifer* specimens, stained by immunohistochemistry with DAPI (white). Yellow arrow heads show examples of pSmad1/5/9 and Runx2-positive cells. Dorsal-spine bones are outlined in orange. All panels except the left-most panel represent magnified views of the area enclosed by the yellow dashed box in the left-most panel. The left-most picture is a confocal Z-stack image, and the others are optical sections obtained by confocal microscopy.

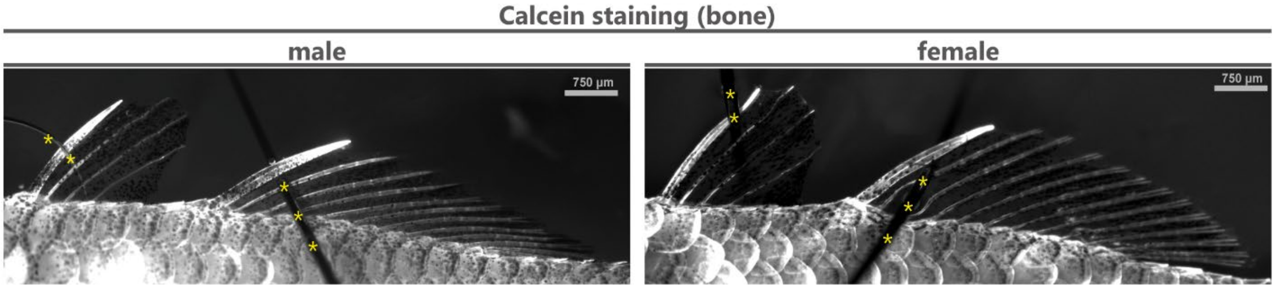

Supplementary Figure 8. Fin bone morphology in the dorsal fin of male and female *Melanotaenia praecox*.

Calcein staining in young adult *M. praecox* showing bone morphology in the dorsal fins (male, $n = 3$; female, $n = 3$). Yellow asterisks indicate the tungsten needles that were used to spread their dorsal fins.

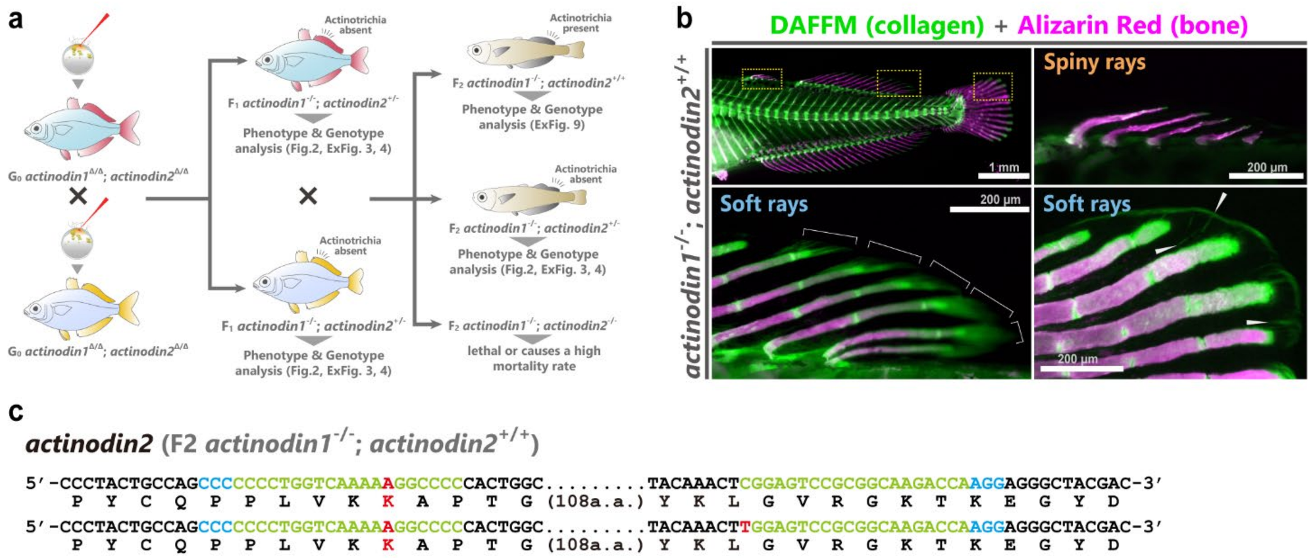

Supplementary Figure 9. Overview of CRISPR/Cas9 mutagenesis workflow and actinotrichia condition of *actinodin1* knockout-only *Melanotaenia praecox*.

a Schematic illustration of the generation of *actinodin1* (*and1*) and *actinodin2* (*and2*) knockout fish. **b** Actinotrichia distribution and spiny- and soft-ray bone morphology in the *and1*$^{-/-}$/*and2*$^{+/+}$ knockout fish. Actinotrichia were labeled with DAFFM DA (green), and the spiny- and soft-ray bones were labeled with alizarin red (magenta). White brackets and arrows indicate actinotrichia ($n = 1$). All panels except the top-left panel represent magnified views of the area enclosed by the yellow dashed box in the top-left panel. **c** Sequences of *actinodin1*$^{-/-}$/*actinodin2*$^{+/+}$ knockout fish, showing a mutation spectrum revealed by Sanger sequencing. Sequences in blue and green indicate the PAM and the recognition sequence for the sgRNA target, respectively. a.a., amino acids.

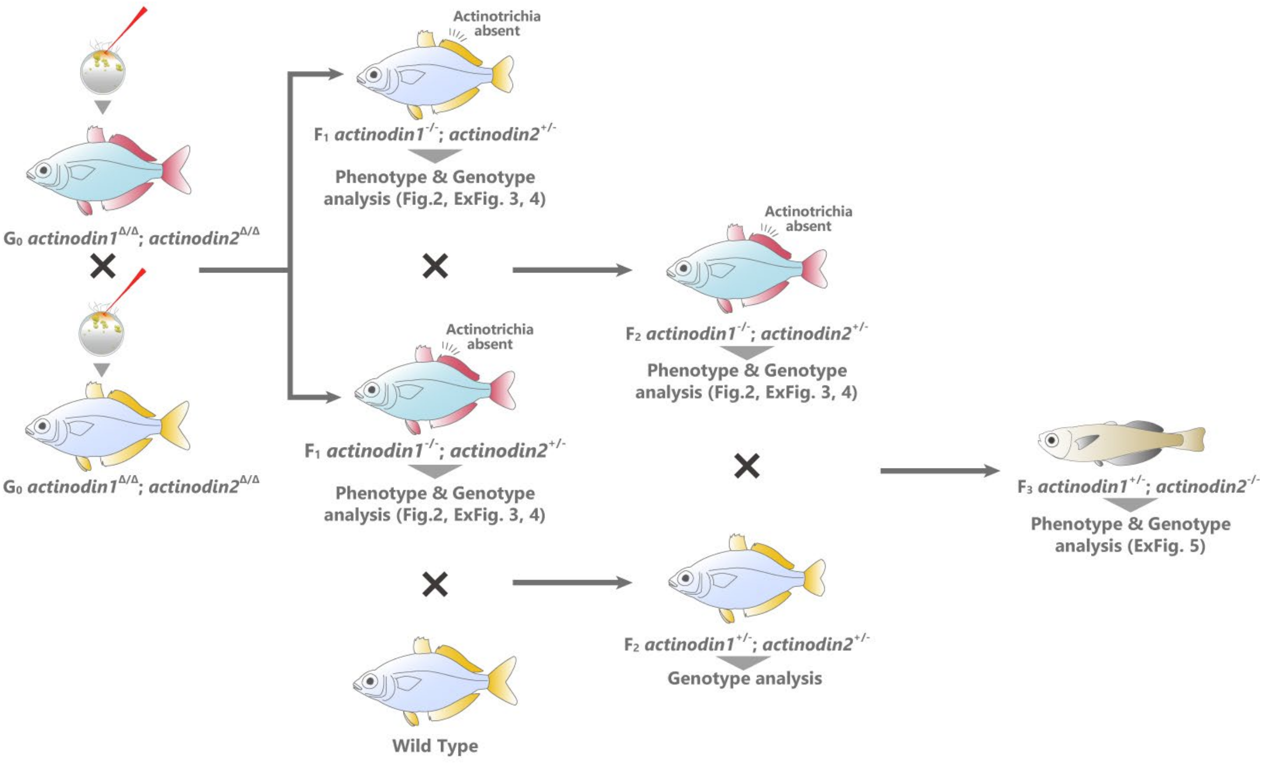

Supplementary Figure 10. Schematic illustration of the obtaining the actinodin1$^{+/-}$/actinodin2$^{-/-}$ knockout fish.
