# Supplementary material for: Actinotrichia-independent developmental mechanisms of spiny rays facilitate the morphological diversification of Acanthomorpha fish fins
Source: Nat Commun. 2026 Feb 14;17:2775. doi: 10.1038/s41467-026-69180-y (PMC13018208; doi:10.1038/s41467-026-69180-y)
Supplement: Supplementary file 2 — Reporting Summary [file 41467_2026_69180_MOESM2_ESM.pdf]

nature portfolio

Corresponding author(s): Kazuhide Miyamoto

Last updated by author(s): Jan 14, 2026

Reporting Summary

Nature Portfolio wishes to improve the reproducibility of the work that we publish. This form provides structure for consistency and transparency in reporting. For further information on Nature Portfolio policies, see our Editorial Policies and the Editorial Policy Checklist.

Statistics

For all statistical analyses, confirm that the following items are present in the figure legend, table legend, main text, or Methods section.

n/a	Confirmed	
☐	☒	The exact sample size (*n*) for each experimental group/condition, given as a discrete number and unit of measurement
☒	☐	A statement on whether measurements were taken from distinct samples or whether the same sample was measured repeatedly
☒	☐	The statistical test(s) used AND whether they are one- or two-sided<br>*Only common tests should be described solely by name; describe more complex techniques in the Methods section.*
☒	☐	A description of all covariates tested
☒	☐	A description of any assumptions or corrections, such as tests of normality and adjustment for multiple comparisons
☒	☐	A full description of the statistical parameters including central tendency (e.g. means) or other basic estimates (e.g. regression coefficient) AND variation (e.g. standard deviation) or associated estimates of uncertainty (e.g. confidence intervals)
☒	☐	For null hypothesis testing, the test statistic (e.g. *F*, *t*, *r*) with confidence intervals, effect sizes, degrees of freedom and *P* value noted<br>*Give P values as exact values whenever suitable.*
☒	☐	For Bayesian analysis, information on the choice of priors and Markov chain Monte Carlo settings
☒	☐	For hierarchical and complex designs, identification of the appropriate level for tests and full reporting of outcomes
☒	☐	Estimates of effect sizes (e.g. Cohen's *d*, Pearson's *r*), indicating how they were calculated

Our web collection on statistics for biologists contains articles on many of the points above.

Software and code

Policy information about availability of computer code

Data collection	Processing microscopy image: LAS X Office v.1.4.28982.7 (Leica), LAS AF (Leica)<br>Processing microscopy image: LuxProcessor v.3.12.0 (Luxendo)<br>Reconstructing Computational Tomography (CT) images: coneCTexpress v.2.6.3.0 or v.2.7.2.0 (White Rabbit Corp.),<br>Processing CT image: Molcer v.1.8.5.1 (White Rabbit Corp.)
Data analysis	Measurement of standard Length of each samples: LAS X Office v.1.4.28982.7 (Leica)

For manuscripts utilizing custom algorithms or software that are central to the research but not yet described in published literature, software must be made available to editors and reviewers. We strongly encourage code deposition in a community repository (e.g. GitHub). See the Nature Portfolio guidelines for submitting code & software for further information.

Data

Policy information about availability of data

All manuscripts must include a data availability statement. This statement should provide the following information, where applicable:

- Accession codes, unique identifiers, or web links for publicly available datasets
- A description of any restrictions on data availability
- For clinical datasets or third party data, please ensure that the statement adheres to our policy

All micro-CT datasets generated in this study are available at Figshare under https://doi.org/10.6084/m9.figshare.30744728. Access to unique biological materials,

including mutant lines of Melanotaenia praecox, is available from the corresponding author upon reasonable request. Some wild-caught specimens of Stephanolepis cirrhifer are subject to local collection and transfer regulations and therefore cannot be freely distributed.

Research involving human participants, their data, or biological material

Policy information about studies with human participants or human data. See also policy information about sex, gender (identity/presentation), and sexual orientation and race, ethnicity and racism.

Reporting on sex and gender	Not applicable
Reporting on race, ethnicity, or other socially relevant groupings	Not applicable
Population characteristics	Not applicable
Recruitment	Not applicable
Ethics oversight	Not applicable

Note that full information on the approval of the study protocol must also be provided in the manuscript.

Field-specific reporting

Please select the one below that is the best fit for your research. If you are not sure, read the appropriate sections before making your selection.

- [x] Life sciences
- [ ] Behavioural & social sciences
- [ ] Ecological, evolutionary & environmental sciences

For a reference copy of the document with all sections, see nature.com/documents/nr-reporting-summary-flat.pdf

Life sciences study design

All studies must disclose on these points even when the disclosure is negative.

Sample size	No statistical methods were used to predetermine sample size. The sample size was chosen empirically to provide sufficient data for comparison.
Data exclusions	No data were excluded
Replication	All attempts of replication were successful.
Randomization	Melanotaenia praecox larvae were stages and then randomly allocated into control or experimental groups.
Blinding	Investigation were not blinded during data collection and/or analysis.

Reporting for specific materials, systems and methods

We require information from authors about some types of materials, experimental systems and methods used in many studies. Here, indicate whether each material, system or method listed is relevant to your study. If you are not sure if a list item applies to your research, read the appropriate section before selecting a response.

Materials & experimental systems

n/a	Involved in the study	
[ ]	[x]	Antibodies
[x]	[ ]	Eukaryotic cell lines
[x]	[ ]	Palaeontology and archaeology
[ ]	[x]	Animals and other organisms
[x]	[ ]	Clinical data
[x]	[ ]	Dual use research of concern
[x]	[ ]	Plants

Methods

n/a	Involved in the study	
[x]	[ ]	ChIP-seq
[x]	[ ]	Flow cytometry
[x]	[ ]	MRI-based neuroimaging

Antibodies

Antibodies used	Primary<br>laminin (1:50, Sigma-Aldrich #L9393)<br>TP63 (1:50, Abcam, #ab735)<br>Runx2 (1:50, Santa Cruz Biotechnology, #sc-101145)

	pSmad1/5/9 (1:100, Cell Signaling Technology, #13820)<br>anti-digoxigenin-AP Fab fragments (1:3000, Roche #11093274910)<br><br>Secondary<br>Alexa Fluor 488 goat anti-rabbit (1:500, Invitrogen, #A-11008)<br>Alexa Fluor 488 goat anti-mouse (1:500, Invitrogen, #A-11001)<br>Alexa Fluor 594 goat anti-rabbit (1:500, Invitrogen, #A-11012)<br>Alexa Fluor 594 goat anti-mouse (1:500, Invitrogen, #A-11032)
Validation	Primary<br>laminin (1:50, Sigma-Aldrich #L9393): https://www.sigmaaldrich.com/JP/ja/product/sigma/l9393?srsltid=AfmBOopxATkRnyAvwkvsECzeYUEMxRsQIazmnHLYzLpXjam3WdwBe7g4<br><br>TP63: https://www.abcam.co.jp/search?gclsrc=aw.ds&gad_source=1&gad_campaignid=23242853952&gbraid=0AAAAADmtO8ac0ww_-p1BbOxBfaeDhJqVG&gclid=CjwKCAiAmp3LBhAkEiwAJM2JUBDLlfSnfD8K4StPBZSAHXAobtK5Ug_KSgV342Vwa3mkEDOREpad1RoC4zYQAvD_BwE#f-adcategorytype=Primary%20Antibodies&sortCriteria=relevance&q=p63<br><br>Runx2: https://www.scbt.com/ja/p/runx2-antibody-27-k?srsltid=AfmBOookgjfX7LBtgTRGovg3UeQ-FRlgVZtWfdKAXazseL7c0OEzV7L8<br>pSmad1/5/9): https://www.cellsignal.com/products/primary-antibodies/phospho-smad1-ser463-465-smad5-ser463-465-smad9-ser465-467-d5b10-rabbit-monoclonal-antibody/13820?srsltid=AfmBOopkpefUCFO1gOA9jASUs9zRENPWbU-PNuodJvk2zl74PDiISfMt<br><br>anti-digoxigenin-AP Fab fragments : https://www.sigmaaldrich.com/JP/ja/product/roche/11093274910?srsltid=AfmBOoq76Az8zdSe5e7TTBqm-NDxUClNVJU3GSbBl0pwn0QEZJEDU7IJ

Animals and other research organisms

Policy information about studies involving animals; ARRIVE guidelines recommended for reporting animal research, and Sex and Gender in Research

Laboratory animals	Melanotaenia praecox were hatched and maintained in a laboratory at Tohoku University. Adults were held in 1-L or 3-L tanks, at ~28 °C, a pH slightly greater than 7.0, and under a light:dark cycle of 14:10 h. Hatched larvae were transferred to a 250-mL rearing tank, at a density of 1–15 individuals per tank. Adults were fed live brine shrimp once or twice daily. Depending on the progeny size, larvae were fed live Paramecium at least once every 2 days, and/or brine shrimp at least once daily. Removal of dead larvae and excreta was performed as required. After the juvenile stage, the progenies were moved to larger tanks (≥250 mL). When observing the dorsal fin bone morphologies of young male and female fish (male, n = 3; female, n = 3), whose sexes are distinguishable by their body coloration, we did not find any significant differences in the fin bone morphologies between males and females at this stage. Furthermore, we did not have a reliable technique to distinguish the sex of larval and juvenile M. praecox using PCR-based methods or other investigations, and thus did not distinguish the sex of the larval and juvenile specimens in our experiments.
Wild animals	Larvae of Stephanolepis cirrhifer were captured at Shoubutahama Fishing Harbor in Miyagi, Japan (38°16′55″ N, 141°03′41″ E). Fish identification. Although sex chromosome system and sexual dimorphisms in the adult second dorsal fin have been reported in this species, we did not distinguish the sex of our specimens because a reliable PCR-based genotyping method has not yet been established. According to Kwon et al. (2021), sexual maturation in this species occurs at a total length of approximately 11.7 cm. The specimens used in our experiments were all significantly smaller than this size and would thus not have been expected to have developed sexual dimorphisms. Furthermore, sexual dimorphisms of the dorsal spine have not been reported. Taken together, we concluded that distinguishing the sex of our S. chirrifer specimens was unnecessary.
Reporting on sex	Sex was specified only for adult Melanotaenia praecox used for CT analysis; for all other specimens, sex was not determined.
Field-collected samples	Wild-caught specimens of Stephanolepis cirrhifer were collected under the appropriate local permits and in accordance with relevant regulations. Melanotaenia praecox specimens were laboratory-reared and not collected from the field.
Ethics oversight	All animal experiments were conducted in accordance with the guidelines and regulations for animal care and use at Tohoku University Animal Research Committee (permit number 2022LsA-002-10). The study was carried out in compliance with ARRIVE guidelines.

Note that full information on the approval of the study protocol must also be provided in the manuscript.

Plants

Seed stocks	*Report on the source of all seed stocks or other plant material used. If applicable, state the seed stock centre and catalogue number. If plant specimens were collected from the field, describe the collection location, date and sampling procedures.*
Novel plant genotypes	*Describe the methods by which all novel plant genotypes were produced. This includes those generated by transgenic approaches, gene editing, chemical/radiation-based mutagenesis and hybridization. For transgenic lines, describe the transformation method, the number of independent lines analyzed and the generation upon which experiments were performed. For gene-edited lines, describe the editor used, the endogenous sequence targeted for editing, the targeting guide RNA sequence (if applicable) and how the editor was applied.*
Authentication	*Describe any authentication procedures for each seed stock used or novel genotype generated. Describe any experiments used to assess the effect of a mutation and, where applicable, how potential secondary effects (e.g. second site T-DNA insertions, mosiacism, off-target gene editing) were examined.*
